# Hyper-androgenemia and obesity in early-pubertal girls

**DOI:** 10.1007/s40618-022-01797-4

**Published:** 2022-04-12

**Authors:** T. Durá-Travé, F. Gallinas-Victoriano

**Affiliations:** 1 Department of Pediatrics, School of Medicine, University of Navarra, Avenue Irunlarrea, 4, 31008 Pamplona, Spain; 2 Department of Pediatrics, Navarra Hospital Complex, Pamplona, Spain; 3 Navarrabiomed (Biomedical Research Center), Pamplona, Spain

**Keywords:** Androgen, Girls, Hormone profile, Obesity comorbidities, Peri-pubertal

## Abstract

**Purpose:**

The aim of this study was to examine the hormonal profile in early-pubertal girls with obesity. We hypothesized that these patients might already present hormonal alterations with POCS-like features.

**Methods:**

Cross-sectional study in a sample of 283 peri-pubertal girls (prepubertal and early-puberty subgroups), aged 6.1–12.0 years, diagnosed with obesity (BMI-SDS > 2.0, 97th percentile), so-called obesity group. They all underwent clinical examination and blood testing for hormonal measurements (leptin, TSH, FT4, IGF-1, IGFBP3, prolactin, insulin, FSH, LH, estradiol, ACTH, cortisol, 17-OH-P, DHE-S, androstenedione, testosterone and free testosterone). A control group was recruited: 243 healthy girls, aged 6.3–12.1 years, with normal BMI status.

**Results:**

Prepubertal girls with obesity had significantly higher values (*p* < 0.05) for BMI-SDS, leptin, insulin and HOMA-IR levels than control group. Early-pubertal girls with obesity also had significantly higher values (*p* < 0.05) for BMI-SDS, leptin, IGF-1, IGFBP3, insulin and HOMA-IR, LH, ratio LH/FSH, ACTH, DHE-S, androstenedione, testosterone and free testosterone levels than control group. In early-pubertal girls with obesity (not prepubertal girls), there was a positive correlation (*p* < 0.01) between leptin levels with LH, androstenedione and testosterone, and HOMA-IR with LH and testosterone levels. There was also a positive correlation (*p* < 0.01) between IGF-1 levels with LH, androstenedione, DHE-S and testosterone; and LH levels with testosterone.

**Conclusion:**

The results obtained support our hypothesis that an abnormal hormonal profile with POCS-like features can already be detected (insulin resistance and hyperinsulinemia, increased secretion of LH and ACTH, and overproduction of ovarian and adrenal androgens) in early-pubertal girls with obesity.

## Introduction

Childhood obesity (children and adolescents) has progressed steadily in most countries in recent decades [1, 2]. In addition, childhood obesity is related to comorbidities potentially harmful to health condition, including hypertension, dyslipidemia, impaired glucose metabolism, obstructive sleep apnea, and non-alcoholic fatty liver disease [3–5]. In fact, it is currently considered as a public health problem of great importance given its social, economic and health repercussions [6].

On the other hand, several observational studies have revealed that excess adiposity could influence various aspects of pubertal development, such as the timing of pubertal onset (although this hypothesis remains controversial), or the hormonal parameters during puberty [7]. For example, peri-pubertal obesity in girls has been associated with hyper-androgenemia in some [8, 9], although not all, studies [10]; despite this, the mechanism underlying this relationship remains uncertain. In addition, it could be said that the sources of androgen overproduction (ovarian vs. adrenal) in overweight pubertal girls are still uncertain [11]. Furthermore, the association between peri-pubertal obesity and hyper-androgenemia could have a potential role in the genesis of polycystic ovarian syndrome (PCOS) during adolescence or later in life [12, 13]. In fact, the PCOS is considered by various authors as a comorbidity of pediatric obesity [14, 15], and this would justify the interest in monitoring the hormonal profile during the different stages of puberty in these girls, especially in early puberty, to take possible preventive measures.

The aim of this study was to examine the hormonal profile in a group of peri-pubertal girls with obesity. We hypothesized that girls with obesity at early puberty might already present an abnormal hormonal profile with POCS-like features.

## Materials and methods

### Participants

This is a cross-sectional study (convenience sample) conducted in a sample of 283 peri-puberal girls, aged 6.1–12.0 years, and diagnosed with obesity (BMI-SDS > 2.0, 97th percentile) or obesity group. They all underwent clinical examination and blood testing for hormonal measurements: leptin, thyrotropin (TSH), free thyroxine (FT4), insulin-like growth factor-1 (IGF-1), IGF-binding protein type 3 (IGFBP3), prolactin (PL), insulin, follicle-stimulating hormone (FSH), luteinizing hormone (LH), estradiol, adrenocorticotrophic hormone (ACTH), cortisol, 17-hidroxyprogesterone (17-OH-P), dehydroepiandrosterone sulfate (DHE-S), androstenedione, total testosterone (T) and free testosterone (FT) in the Pediatric Endocrinology Unit of the Navarra Hospital Complex (Pamplona, Spain) in the period January 2013–December 2021.

In addition to that, these parameters (clinical examination and hormonal measurements) were determined in a control group that consisted of 243 healthy girls, aged 6.3–12.1 years, with normal nutritional status: BMI-SDS ranging from − 1.0 (15th percentile) to + 1.0 (85th percentile). These participants came from external consultations of the different pediatric subspecialties.

Breast development was assessed by both inspection and palpation and pubertal stage was determined in each participant according to Tanner’s criteria. In this way, the participants were classified in two different subgroups: prepubertal subgroup (Tanner breast stage I) and early-puberty subgroup (Tanner breast stages II). All participants were pre-menarcheal girls.

All participants included in the study were Caucasian individuals with no previously detected chronic pathologies that might affect growth, body composition, food ingestion or physical activity. Girls who had a history of virilization (hirsutism, clitoromegaly, etc.), premature adrenarche or precocious puberty were excluded.

Adequate information of the proceedings and potential implications was delivered to the parents and/or legal guardians, and the corresponding consent was required prior to the inclusion in this study in all cases. The study was presented and subsequently approved after the evaluation of the Ethics Committee for Human Investigation at our institution (in line with the ethical standards stated in the Declaration of Hensinki 1964 and later amendments).

### Clinical examination

Weight and height measurements were made in underwear while barefoot. Weight was measured using an Año-Sayol scale (reading interval 0 to 120 kg and a precision of 100 g), and height was measured using a Holtain wall stadiometer (reading interval 60 to 210 cm, precision 0.1 cm). The SDS values for the BMI were calculated using the epidemiologic data contained within the program Aplicación Nutricional, from the Spanish Society of pediatric gastroenterology, hepatology and nutrition (Sociedad Española de Gastroenterología, Hepatología y Nutrición Pediátrica, available at http://www.gastroinf.es/nutritional/). The graphics from Ferrández et al. (Centro Andrea Prader, Zaragoza 2002) used as reference charts [16].

### Hormonal measurements

All hormonal determinations (leptin, TSH, FT4, IGF-1, IGFBP3), PL, insulin, FSH, LH, estradiol, ACTH, cortisol, 17-OH-P, DHE-S, androstenedione, T and FT were made after blood sample collection under basal fasting conditions (between 8:00 and 9:00 h after an overnight fast).

Hormonal measurements, when appropriate, were quantified by radio immuno-assay (Immuno Diagnostic System, Bolden, UK), enzyme-linked immunosorbent assay (Immulite analyzer, DPC Biermann, Bad Nauheim, Germany) or highly sensitive chemiluminescence immuno-assays (LIAISON Assay, Diasorin, Dietzenbach, Germany). Homeostasis model-assessment (HOMA-IR) indexes were calculated from fasting glucose and insulin concentrations (glucose levels in mmol × insulin in μUml/L/22.5).

### Statistical analysis

Results are displayed as means (M) with the corresponding confidence intervals (95% CI) or median and interquartile ranges. Mann–Whitney *U* test or Student’s *t* test was used to compare mean or median values in the variables recorded within groups and subgroups. Pearson's test was used to quantify the degree of linear association between quantitative variables. Statistical analyses were performed using the program Statistical Packages for the Social Sciences version 20.0 (Chicago, IL, USA). Statistical significance was accepted when *p* value was < 0.05.

## Results

Table 1 shows and compares the mean values for the clinical characteristics (age and BMI-SDS) and hormonal determinations (leptin, TSH, FT4, IGF-1, PL, insulin and HOMA-IR) in prepubertal and early-puberty subgroups according to BMI status (control and obesity groups).

**Table 1:** Mean clinical characteristics (age and BMI-SDS) and hormonal measurements (leptin, TSH, FT4, IGF-1, IGFBP3, PL, insulin and HOMA-IR) in prepubertal and early subgroups according to the BMI status (M, 95% CI)

	Control group (*n* = 132)	Obesity group (*n* = 165)	*p* values*
*Prepubertal subgroup*			
Age (years)	8.1 (7.8–8.3)	8.1 (7.8–8.3)	0.864
BMI-SDS	−0.02 (−0.12/ + 0.08)	3.4 (3.2–3.6)	0.001
Leptin (μg/L)	6.8 (4.1–9.6)	26.6 (24.3–29.0)	0.001
TSH (mU/L)	2.0 (1.8–2.2)	2.1 (2.0–2.3)	0.301
FT4 (ng/dL)	1.0 (0.9–1.0)	1.0 (0.9–1.1)	0.450
IGF-1 (μg/L)	182.1 (168.9–195.4)	182.2 (170.7–193.6)	0.994
IGFBP3 (μg/L)	4997.9 (4748.3–5247.4)	5238.1 (4910.1–5567.2)	0.235
PL (μg/L)	11.8 (10.1–13.5)	11.3 (9.5–13.1)	0.681
Insulin (mU/L)	7.2 (5.6–8.8)	14.4 (13.1–15.8)	0.001
HOMA-IR	1.6 (1.2–1.9)	3.0 (2.7–3.3)	0.001

	Control group (*n* = 111)	Obesity group (*n* = 118)	*p* values*
*Early puberty subgroup*			
Age (years)	10.5 (10.0–11.0)	10.4 (9.9–10.8)	0.831
BMI-SDS	0.10 (−0.01/ + 0.19)	3.3 (3.1–3.5)	0.001
Leptin (μg/L)	8.8 (4.8–12.9)	45.2 (41.1–49.3)	0.001
TSH (mU/L)	2.2 (2.1–2.3)	2.3 (2.1–2.6)	0.761
FT4 (ng/dL)	1.02 (1.0–1.04)	1.01 (0.99–1.03)	0.823
IGF-1 (μg/L)	260.6 (236.5–284.8)	367.4 (311.5–423.4)	0.001
IGFBP3 (μg/L)	5636.4 (5315.9–5956.8)	6509.1 (5822.7–7195.4)	0.009
PL (μg/L)	10.0 (8.5–11.4)	12.2 (9.6–14.8)	0.544
Insulin (mU/L)	11.2 (9.1–13.3)	25.3 (22.4–28.1)	0.001
HOMA-IR	2.4 (1.9–2.8)	4.9 (4.4–5.3)	0.001

*Student’s *t* test

In prepubertal subgroup, girls with obesity had significantly higher values (*p* < 0.05) for BMI-SDS, leptin and insulin levels, and HOMA-IR index than control group, and there were no significant differences in age, TSH, FT4, IGF-1, IGFBP3, and PL levels among both groups. In early-puberty subgroup, girls with obesity had significantly higher values (*p* < 0.05) for BMI-SDS, leptin, IGF-1, IGFBP· and insulin levels, and HOMA-IR index than control group, and there were no significant differences in age, TSH, FT4 and PL levels among both groups.

Table 2 shows and compares the mean values for the hormonal determinations (FSH, LH, estradiol, ACTH, cortisol, 17-OH-P, DHE-S, androstendiona, T and FT) in prepubertal and early-puberty subgroups in accordance to BMI status (control and obesity groups).

**Table 2:** Mean hormonal determinations (FSH, LH, ratio LH/FSH, estradiol, ACTH, cortisol, 17-OH-P, DHE-S, androstendiona, T and FT) in prepubertal and early subgroups according to the BMI status (M, 95% CI)

	Control group (*n* = 132)	Obesity group (*n* = 165)	*p* values*
*Prepubertal subgroup*			
FSH (UI/L)	1.5 (1.3–1.6)	1.6 (1.0–2.2)	0.754
LH (UI/L)	0.05 (0.04–0.06)	0.050 (0.03–0.06)	0.275
ratio LH/FSH	0.03 (0.03–0.04)	0.04 (0.03–0.04)	0.314
Estradiol (pg/mL)	9.1 (8.5–9.7)	9.5 (9.2–9.8)	0.126
ACTH (ng/L)	19.8 (15.9–23.6)	22.3 (19.3–25.3)	0.299
Cortisol (μg/dL)	8.6 (7.7–0.6)	8.1 (7.2–9.0)	0.421
17-OH-P (μg/L)	0.8 (0.6–1.0)	0.80 (0.7–0.8)	0.435
DHE-S (μg/dL)	61.0 (53.3–68.7)	71.7 (64.2–79.2)	0.253
Androstenedione (μg/L)	0.4 (0.3–0.5)	0.4 (0.3–0.4)	0.752
Testosterone (ng/mL)	0.1 (0.09–0.11)	0.11 (0.10–0.12)	0.674
FT (ng/L)	0.33 (0.27–0.39)	0.40 (0.32–0.48)	0.220

	Control group (*n* = 111)	Obesity group (*n* = 118)	*p* values*
*Early puberty subgroup*			
FSH (UI/L)	3.7 (3.3–4.1)	3.8 (3.5–4.2)	0.529
LH (UI/L)	0.7 (0.5–0.9)	2.5 (2.0–2.9)	0.001
ratio LH/FSH	0.19 (0.15–0.24)	0.6 (0.5–0.7)	0.001
Estradiol (pg/mL)	24.6 (17.6–31.5)	31.0 (24.4–37.5)	0.310
ACTH (ng/L)	18.1 (14.9–21.1)	29.6 (24.8–34.3)	0.003
Cortisol (μg/dL)	9.2 (6.8–11.5)	11.5 (9.6–13.3)	0.389
17-OH-P (μg/L)	0.8 (0.7–1.0)	0.9 (0.7–1.1)	0.318
DHE-S (μg/dL)	69.6 (61.0–78.3)	143.8 (130.6–156.9)	0.001
Androstenedione (μg/L)	0.6 (0.5–0.7)	1.4 (1.2–1.6)	0.001
Testosterone (ng/mL)	0.14 (0.10–0.18)	0.23 (0.21–0.25)	0.001
FT (ng/L)	0.6 (0.4–0.7)	1.3 (1.1–1.3)	0.001

*Student’s *t* test

In the prepubertal subgroup, there were no significant differences in any hormonal measurements among both groups. In early-puberty subgroup, girls with obesity had significantly higher values (*p* < 0.05) for LH, ACTH, androstendiona, T and FT levels than control group, and there were no significant differences in age, FSH, estradiol, cortisol and 17-OH-P levels among both groups.

In control group, early-puberty subgroup girls had significantly higher values (*p* < 0.05) for age, IGF-1, IGFBP3, insulin, HOMA-IR, FSH, LH, ratio LH/FSH, estradiol, androstenedione, T and FT levels than prepubertal subgroup, and there were no significant differences in BMI-SDS, leptin, TSH, FT4, PL, ACTH, cortisol, 17-OH-P, and DHE-S levels among both subgroups.

In obesity group, early-puberty subgroup girls had significantly higher values (*p* < 0.05) for age, leptin, IGF-1, IGFBP3, insulin, HOMA-IR, FSH, LH, ratio LH/FSH, estradiol, ACTH, DHE-S, androstenediona, T and FT levels than prepubertal subgroup, and there were no significant differences in BMI-SDS, TSH, FT4, PL, cortisol and 17-OH-P levels among both subgroups.

In early-pubertal girls with obesity (not prepubertal girls), there was a positive correlation (*p* < 0.01) between BMI and HOMA-IR (*r* = 0.649), leptin (*r* = 0.627) and T (*r* = 0.424); as well as between leptin and LH (*r* = 0.464), androstenedione (*r* = 0.366) and T (*r* = 0.454) levels, and between HOMA-IR and LH (*r* = 0.489) and T (*r* = 0.416). In addition, there was a positive correlation (*p* < 0.01) between IGF-1 and LH (*r* = 0.689), androstenedione (*r* = 0.497), DHE-S (*r* = 0.486) and T (*r* = 0.404) levels, as well as between LH and T (*r* = 0.676) levels, and between T and FT (*r* = 0.768) levels.

## Discussion

This study features that hormonal profile in peri-pubertal girls with obesity is characterized by hyperinsulinemia and higher HOMA-IR index, especially evident in early puberty, compared to control group. Furthermore, in early puberty (not prepubertal girls), these girls with obesity showed higher levels of LH, ACTH, and androgens (DHEA-S, androstenedione, T and FT) than non-obese girls.

Insulin resistance and compensatory hyperinsulinemia are physiological during the peri-pubertal period, but insulin resistance may be exaggerated especially in peri-pubertal girls with obesity -as occurred in this study-, and is a frequent obesity-related comorbidity, as well as a risk factor for atherosclerotic cardiovascular disease and type 2 diabetes mellitus [4, 9, 15]. In this case, the analysis of this finding is quite interesting because experimental studies have revealed a relationship between hyper-androgenemia and hyperinsulinemia in peri-pubertal girls with obesity. As an example, insulin appears to stimulate androgen production in the ovarian theca cells even in the absence of LH, as well as to promote excessive androgen production by the adrenal glands. Hyperinsulinemia decreases hepatic production of sex hormone-binding globulin (SHBG) resulting in increased androgen bioavailability (i.e., increased free testosterone), and these abnormalities appear to reverse with weight loss [17]. Even more, it appears that insulin also directly up-regulates 17-hydroxysteroid dehydrogenase gene expression and activity, stimulating testosterone formation from androstenedione in the ovaries and zona reticularis of the adrenal cortex [18]. In the present study, as several authors have previously described [8, 9, 12], we found a significant correlation between insulin resistance (HOMA-IR index) and testosterone (total and free fraction) in early-pubertal girls with obesity. Although the pathophysiology of hyper-androgenemia in peri-pubertal girls with obesity is yet unclear, insulin resistance with compensatory hyperinsulinemia probably plays a key role [9]. Therefore, although prepubertal girls with obesity did not show hyper-androgenism in this study, they should be regularly monitored because they already had higher insulin and HOMA-IR levels than the control group [19]. The application of an extensive combined dietary behavioral–physical activity intervention helps improve cardiovascular risk factors, such as hypertension, dyslipidemia, and insulin resistance [20]; and some authors have reported that androgen levels improve after sustained weight loss [14, 17]. However, additional studies are required to determine if weight loss and/or pharmacological treatment could improve insulin resistance and decrease androgen levels in these patients.

Under normal conditions, androgens and androgen precursors are normally secreted by both the ovaries and the adrenal glands in response to their respective trophic hormones, LH and ACTH. Approximately half of testosterone is produced by direct secretion of the ovaries and adrenal glands, while the other half is produced by peripheral conversion of circulating androstenedione mainly in liver, skin and fat [21]. In fact, obesity itself increases androgen production and peripheral conversion of androstenedione to testosterone in adipose tissue. The factors regulating these conversions are unclear, although insulin, IGF-1, and leptin have been suggested as determinants in stimulating testosterone synthesis in fat [22, 23]. In our study, we found a significant correlation between BMI-SDS and leptin with androstenedione and testosterone levels, suggesting, as some authors support, that excess adipose tissue appears to be an important factor to androgen excess in these patients [19, 21, 24, 25].

Total IGF-1 and IGFBP3 concentrations in subjects with obesity are mildly elevated, as we observed in this study, but free IGF-1 levels are increased. The latter may reflect reductions in circulating IGBP-1 and IGFB2, which are suppressed by insulin and correlate inversely with insulin sensitivity [26]. Together with nutrients excess, the increase in free IGF-1 levels may accelerate linear growth and bone age in peri-pubertal period and, with hyperinsulinemia, it would contribute to adrenal and ovarian androgen production [27]. In fact, we also found in early-pubertal girls with obesity a significant correlation between IGF-1 and androgen levels (DHE-S, androstenedione and testosterone). Additional studies are needed to corroborate the presumed relationship between IGF-1 and overproduction of androgens.

In compliance with several studies [8, 9], we also found that the hormonal profile in early-pubertal girls with obesity showed higher levels of morning LH concentrations than non-obese girls, as well as a significant correlation between morning LH and testosterone levels. This association suggests that LH is a permissive factor for androgen excess, since LH is the proximal stimulus for androgen production by ovarian theca cells. And, in susceptible girls, free androgens would interfere with normal negative feedback mechanism at the GnRH pulse generator, increasing both GnRH and LH pulses and the LH/FSH ratio, thereby enhancing thecal androgen production [28–31]. This means, it seems that not only obesity or hyperinsulinemia but also other factors, such as pubertal levels of gonadotropins, would be included in the pathogenesis of hyper-androgenemia. In fact, several authors have considered LH, followed by fasting insulin, as the best predictor of hyper-androgenemia in obese peri-pubertal girls [8, 21].

Interestingly, the different authors who have studied hyper-androgenemia throughout pubertal transition in girls with obesity have obviated morning ACTH levels [8, 19, 32]. However, in this study, we found that early-pubertal girls with obesity showed higher levels of ACTH than non-obese girls. The reticular zone of the adrenal gland functionally resembles the theca cells of the ovary for androgen production, but in response to their trophic hormone (ACTH). Therefore, this eventuality would mean that the adrenal gland appears to be a source of excessive androgen production in early-pubertal girls with obesity (ACTH-dependent adrenal source) and, consequently, a contributor to adiposity-related hyper-androgenemia in these patients. This hypothesis would support the concept that a mixed adrenal and ovarian over-secretion of androgens in early-puberty girls with obesity could be a facilitating factor for the development of hyper-androgenemia [11, 33].

Hyper-androgenemia often has no clinical manifestations during early puberty, but despite its subclinical manifestations, it may represent a precursor of adult PCOS. In fact, different authors consider that resistance to insulin and elevated levels of androgen would be the main causes of polycystic ovarian syndrome [13, 34]. Thus, the association between peri-pubertal obesity and hyper-androgenemia may suggest that girls with obesity are at high risk for future POCS. In fact, several authors have suggested that all adolescent girls with obesity should be asked about possible symptoms of PCOS [12, 14, 29], and some authors have even suggested the possibility of defining a condition of "PCOS secondary to obesity” [35]. Nevertheless, prospective longitudinal research studies will be helpful to understand the natural history of girls considered to be at risk for PCOS. Obviously, our findings evidence that early-puberty girls with obesity display an abnormal hormonal profile with POCS-like features.

An important limitation of our study is the cross-sectional design and, therefore, our findings reflect an association, but do not prove causality. However, the data obtained agree with the following working hypothesis concerning obesity-related hyper-androgenemia: in some obese peri-pubertal girls, excessive IGF-1 and insulin act in synergy with ACTH and LH to stimulate the production of androgens from adrenocortical cells and ovarian theca cells respectively [7]. In addition, SHBG was not measured in this study because our laboratory routinely determines the free fraction of testosterone by highly sensitive chemiluminescence immuno-assays; and therefore in this study, we were unable to verify if SHBG levels were decreased among early-pubertal girls with obesity. On the other hand, data about girls who were “at risk of overweight” (BMI-for-age between 85 and 95th percentile) are not included in this study since they are generally at intermediate risk for obesity-related abnormalities.

In conclusion, the results obtained support our hypothesis that, in early-pubertal girls with obesity, an abnormal hormonal profile with POCS-like features can already be observed. In fact, they display a hormonal profile characterized by endocrinological dysfunction (increased secretion of LH with normal FSH) together with metabolic dysfunction (insulin resistance and hyperinsulinemia) and dysfunction of ovarian/adrenal steroidogenesis (overproduction of ovarian and adrenal androgens). Additional studies are required to determine if weight loss and/or pharmacological treatment could improve both insulin resistance and decrease androgen levels in these patients.

## Data Availability

The datasets generated during and/or analyzed during the current study are available from the corresponding author on reasonable request.

## Hormonal profile in obese peri-pubertal girls

See Tables 3 and 4.

**Table 3:** Clinical characteristics (age and BMI-SDS) and hormonal measurements (leptin, TSH, FT4, IGF-1, IGFBP3, PL, insulin and HOMA-IR) in prepubertal and early subgroups according to the BMI status (median and interquartile range)

	Control group (*n* = 132)	Obesity group (*n* = 165)	*p* values*
*Prepubertal subgroup*			
Age (years)	8.1 (8.0–8.5)	8.2 (7.8–8.5)	0.864
BMI-SDS	−0.02 (−0.12–0.39)	3.3 (2.5–3.9)	0.001
Leptin (μg/L)	6.1 (3.7–10.8)	25.2 (19.2–34.5)	0.006
TSH (mU/L)	1.98 (1.4–2,1)	2.1 (1.5–2.8)	0.328
FT4 (ng/dL)	1.0 (0.9–1.0)	1.0 (0.9–1.1)	0.207
IGF-1 (μg/L)	177.1 (148.2–207.2)	179.0 (149.6–214.1)	0.872
IGFBP3 (μg/L)	4860.1 (4485.3–5387.1)	5090.2 (4453.3–5617.5)	0.628
PL (μg/L)	10.8 (8,0–14.2)	10.3 (8.4–13.9)	0.556
Insulin (mU/L)	6.9 (5.1–9.8)	13.2 (8.9–17.9)	0.001
HOMA-IR	1.5 (1.1–2.3)	2.9 (2.1–4.0)	0.001

	Control group (*n* = 111)	Obesity group (*n* = 118)	*p* values*
*Early puberty subgroup*			
Age (years)	9.9 (9.3–10.5)	10.2 (9.4–10.9)	0.831
BMI-SDS	0.10 (−0.7–0.5)	3.2 (2.3–3.7)	0.001
Leptin (μg/L)	8.5 (6.3–12.2)	43.9 (35.4–55.6)	0.001
TSH (mU/L)	2.1 (1.5–2.7)	2.2 (1.6–2.9)	0.643
FT4 (ng/dL)	1.0 (0.9–1.1)	0.9 (0.8–1.0)	0.682
IGF-1 (μg/L)	256,1 (210.5–312,4)	345,6 (267.2–424.4)	0.378
IGFBP3 (μg/L)	5705.4 (5160.3–6172.5)	6370.2 (5370.6–7457.4)	0.288
PL (μg/L)	9.3 (7.3–13.2)	11.1 (8.1–15.4)	0.210
Insulin (mU/L)	11.8 (6.3–14.6)	22.3 (15.6–31.4)	0.001
HOMA-IR	2.3 (1.5–3.2)	4.5 (3.7–6.9)	0.001

*Mann–Whitney *U* test

**Table 4:** Hormonal determinations (FSH, LH, estradiol, ACTH, cortisol, 17-OH-P, DHE-S, androstendiona, T and FT) in prepubertal and early subgroups according to the BMI status (median and interquartile range)

	Control group (*n* = 132)	Obesity group (*n* = 165)	*p* values*
*Prepubertal subgroup*			
FSH (UI/L)	1.4 (0.8–1.9)	1.5 (0.6–1.9)	0.632
LH (UI/L)	0.04 (0.02–0.07)	0.04 (0.02–0.05)	0.451
Estradiol (pg/mL)	9.1 (6.3–9.9)	9.2 (9.0–9.7)	0.830
ACTH (ng/L)	18.1 (13.5–24.8)	21.9 (15.1–28.2)	0.234
Cortisol (μg/dL)	7.9 (5.3–10.6)	7.4 (5.1–10.9)	0.411
17-OH-P (μg/L)	0.7 (0.5–0.9)	0.7 (0.5–1.1)	0.908
DHE-S (μg/dL)	62.1 (45.9–78.7)	66.6 (43.3–81.45)	0.532
Androstenedione (μg/L)	0.3 (0.3–0.4)	0.3 (0.3–0.4)	0.934
Testosterone (ng/mL)	0.1 (0.06–0.1)	0.1 (0.08–0.1)	0.744
FT (ng/L)	0.3 (0.1–0.5)	0.2 (0.1–0.5)	0.331

	Control group (*n* = 111)	Obesity group (*n* = 118)	*p* values*
*Early puberty subgroup*			
FSH (UI/L)	3.6 (2.5–4.8)	4.2 (2.3–5.3)	0.163
LH (UI/L)	0.4 (0.2–1.1)	2.3 (0,8–3.9)	0.001
Estradiol (pg/mL)	21.6 (14.6–41.1)	28.0 (18.4–33.5)	0.810
ACTH (ng/L)	15.1 (13.9–24.5)	27.7 (20.7–38.1)	0.013
Cortisol (μg/dL)	8.2 (5.6–10.9)	10.0 (7.1–12.9)	0.240
17-OH-P (μg/L)	0.7 (0.5–0.9)	0.9 (0.6–1.2)	0.322
DHE-S (μg/dL)	65.1 (49.3–87.1)	138.2 (97.2–166.9)	0.001
Androstenedione (μg/L)	0.5 (0.3–0.7)	1.3 (0.8–1.7)	0.001
Testosterone (ng/mL)	0.13 (0.09–0.15)	0.21 (0.18–0.285)	0.001
FT (ng/L)	0.5 (0.3–0.9)	1.2 (0.7–1.7)	0.001

*Mann–Whitney *U* test
